# Changing Perspectives on Pediatric Human Papillomavirus (HPV) Vaccination among Dental Students and Residents Reveals Recent Increase in Vaccine Hesitancy

**DOI:** 10.3390/vaccines10040570

**Published:** 2022-04-06

**Authors:** Rebecca Maginot, Carolina Esteves, Karl Kingsley

**Affiliations:** 1 Department of Advanced Education in Pediatric Dentistry, School of Dental Medicine, University of Nevada, 1700 W. Charleston Boulevard, Las Vegas, NV 89106, USA; maginot@unlv.nevada.edu; 2 Department of Clinical Sciences, School of Dental Medicine, University of Nevada, 1700 W. Charleston Boulevard, Las Vegas, NV 89106, USA; estevc1@unlv.nevada.edu; 3 Department of Biomedical Sciences, School of Dental Medicine, University of Nevada, 1001 Shadow Lane, Las Vegas, NV 89106, USA

**Keywords:** human papillomavirus (HPV), vaccination hesitancy, dental student, postgraduate resident

## Abstract

This study was a retrospective analysis of previously collected anonymous survey data regarding vaccine awareness, beliefs, and knowledge among dental (DMD) students and postgraduate (PG) residents. The protocol for this study was approved by the Institutional Review Board (IRB) as exempt. A total of 341 responses were collected from n = 293 DMD students and n = 48 PG residents. Although most respondents agreed that vaccines were necessary, safe, and effective, over the past 4 years (2017–2020) a growing percentage of respondents disagreed. In addition, although most respondents disagreed that there are too many required vaccines, vaccines can make you sick, or are dangerous, a growing percentage of respondents now agreed with these statements. Finally, although most respondents were aware of the HPV vaccine, recently a growing percentage of both students and residents reported they had insufficient information about this vaccine. These results provide novel insights into recent changes in attitudes and beliefs regarding vaccination among this population. Moreover, analysis of these shifts in attitudes and knowledge about HPV vaccination suggests that curricular integration of vaccine research and hesitancy may be needed to answer these questions in a supportive learning environment that fosters critical thinking and evidence-based practice and decision making.

## 1. Introduction

There are ever-changing and evolving messages in the media and social media landscape regarding the value and efficacy of pediatric vaccinations [1,2]. An increasing volume of misinformation and disinformation has led to significant increases in vaccine hesitancy and mistrust even among highly educated populations [3,4]. Successful strategies are needed for healthcare providers to directly address this misinformation and the associated parental vaccination hesitancy in order to improve public health and patient outcomes [5,6,7].

Many of these strategies to address vaccine misinformation and vaccine hesitancy involve communication between healthcare providers and patients, which has become increasingly more important and also more controversial [8,9]. Recent studies have uncovered deeply rooted vaccine hesitancy may be linked to sentiments strongly associated with social influences, including family and close friends, rather than direct engagement or evaluation of medical or scientific information or misinformation [10,11]. However, research has demonstrated that these attitudes and beliefs may indeed be malleable and subject to change following positive and direct communication of health information from trusted healthcare workers and providers [12,13].

Although many studies have evaluated the role of specific healthcare providers, such as pediatricians, in addressing vaccine hesitancy, an increasing body of research has demonstrated that dentists and dental providers may also have significant influence on vaccination acceptance and parental perceptions, particularly in the area of human papillomavirus (HPV) vaccination [14,15,16]. This may be due to the revelation that vaccine hesitancy may be overcome by not a single episode of positive healthcare communication, but rather by repeated interactions with varied healthcare professionals using similar and reinforcing messages, such as vaccine information received from a pediatrician and subsequently reinforced by similar messages from a pediatric dentist [17,18].

Vaccination knowledge, awareness, and acceptance strongly influence provider recommendations, yet relatively few studies have focused on assessing these aspects of dental provider HPV vaccine literacy [19,20]. Optional educational interventions have been shown to increase dental provider willingness to engage in vaccine recommendations and HPV-specific vaccine patient communications; incorporation of this into dental school educational curriculum may be the most effective method to train future dental professionals in these efforts [21,22,23].

However, few studies have focused on the evaluation of HPV vaccination knowledge, awareness, and acceptance among dental students and post-graduate residents to determine which, if any, educational interventions are needed to address any deficiencies or identify any existing forms of vaccine hesitancy among this population [24,25,26]. In addition, recent shifts in social and political discourse regarding vaccines and vaccination may be further influencing the attitudes, awareness, and acceptance of vaccination among this specific population [27,28,29]. Based upon this information, the primary objective of this study was to provide a more current and up-to-date evaluation of these recent trends among dental students and post-graduate residents to thereby identify any differences that might suggest opportunities for educational interventions or training.

## 2. Materials and Methods

### 2.1. Human Subjects Approval

The protocol for this study was reviewed and approved by the University of Nevada, Las Vegas (UNLV) Institutional Review Board (IRB) under protocol number [1762988-2] titled “Retrospective analysis of educational in-class dental student survey” on 8 June 2021. Informed consent for this retrospective study and analysis was waived in accordance with the Basic Health and Human Services (HHS) Policy for the Protection of Human Research Subjects (46.101); Subpart A (b) regarding research involving the use of educational tests (cognitive, diagnostic, aptitude, achievement) in which the subjects cannot be identified directly or through identifiers.

In brief, enrolled dental students and post-graduate dental residents were asked to complete a voluntary survey, following a scheduled curricular module focusing on vaccination, with a specific focus on human papillomavirus (HPV) vaccination and the relevance to dentistry. The brief survey was based upon a previously validated knowledge, awareness, and clinical practice survey as previously described [26,30]. Students or residents were asked to select questions of interest and find peer-reviewed or evidence-based information to support or refute their selected answer choices. Following this in-class discussion and exercise, students were asked to voluntarily turn in their survey and supporting evidence with or without demographic information (age, sex, race, or ethnicity only) but with no specific identifying information regarding the respondents (no student identification numbers or other specific identifiers).

### 2.2. Survey

The full questionnaire consisted of n = 17 total questions, divided between two sections. The first section consisted of eight non-specific vaccine-related questions assessing general knowledge, awareness, perceptions, and clinical practice guidelines. The second section consisted of HPV-specific questions. Four possible responses were available (Disagree, Neutral, Agree, and Not applicable). These questions included:

Section 1: General questions

1. Vaccines are necessary to protect public health
2. There are too many required vaccines
3. Vaccines are generally safe
4. Vaccination can make you sick
5. Some vaccines are dangerous
6. Vaccines are generally effective
7. I follow the ACIP vaccine guidelines for myself
8. I adhere to the vaccine guidelines for my family

Section 2: HPV-specific questions

1. I am aware of a vaccine for human papillomavirus (HPV)
2. HPV vaccination is important for me
3. HPV vaccination is important for (my) spouse/partner
4. HPV vaccination is important for (my) daughter(s)
5. HPV vaccination is important for (my) son(s)
6. I have discussed HPV vaccination with a doctor
7. I do not have enough information about the HPV vaccine
8. I am concerned about possible HPV vaccine side effects
9. I have already received the HPV vaccine

### 2.3. Statistical Analysis

All demographic data were reported as descriptive statistics and differences between categorical variables, such as sex (male, female) and race/ethnicity (white, minority) were evaluated using overall clinic population data and Chi Square analysis using the GraphPad (San Diego, CA, USA) online Chi square calculator, which is appropriate for non-parametric analysis of categorical data. Two-tailed t-tests were used to determine any differences between the average age of study samples compared with the overall age of the cohort (DMD or PGR), which is appropriate for parametric analysis of continuous data.

All responses from each paper survey were input manually into an Excel Spreadsheet. Responses for each question were tabulated and descriptive statistics were compiled for each year of dental students and residents. Any differences in frequency of responses between questions or between dental student and postgraduate resident cohorts were plotted and graphed. Differences in the percentage of responses in specific question categories (e.g., Agree) between the doctor of medicine in dentistry (DMD) or dental students and postgraduate resident (PGR) cohorts were calculated using Chi Square analysis, as described above.

## 3. Results

This voluntary survey was administered to all DMD students over the course of 4 years (n = 327), with n = 293 students completing the survey to yield an approximate 89.6% response rate (Table 1). The overall percentage of males and females in the DMD student population (57.8%, 42.2%, respectively) was not significantly different from the proportion of males and females that completed the survey (54.9% and 45.1%, respectively), *p* = 0.4750. In addition, the proportion of white and minority students in the DMD student population (42.8%, 57.2%, respectively) was also not significantly different from the survey study population (41.6%, 58.4%, respectively), *p* = 0.7674. The average age of respondents was 25.46, which was similar to the overall average age of the overall DMD student population (25.62), *p* = 0.7134.

In addition, the same voluntary survey was administered to all postgraduate residents (PGR) over the same time interval (n = 48), with n = 41 residents completing the survey to yield an approximate 85.4% response rate. The overall percentage of males and females among the PGR population (45.8%, 54.2%, respectively) was not significantly different from the percentage of males and females that completed the survey (46.3%, 53.7%, respectively), *p* = 0.9618. Finally, the proportion of white and minority PGR population (50.0%, 50.0%, respectively) was also not significantly different from the PGR survey study population (51.2%, 48.8%, respectively), *p* = 0.9087. The average age of PGR respondents (26.7 years) was not significantly different than the PGR population (27.1 years), *p* = 0.7749.

To assess the general vaccine-related responses of DMD students and PGR, the survey responses to Questions 1–8 were compiled and descriptive statistics calculated (Table 2). The analysis of these data demonstrated the majority of pro-vaccine responses from DMD students and PGR who agreed with Question 1 about vaccine necessity, which was similar but higher among DMD students (90.1%) compared with PGRs (85.4%), χ^2^ = 0.860, d.f. = 1, *p* = 0.3537. In addition, fewer DMD students agreed with Question 3 about vaccine safety (87.7%) compared with PGRs (97.6%), χ^2^ = 3.541, d.f. = 1, *p* = 0.0599. Some differences were also found among those who agreed with Question 6 about vaccine effectiveness among the cohorts (DMD, 90.1%; PGR 97.6%), χ^2^ = 2.447, d.f. = 1, *p* = 0.1177.

The analysis of these data also demonstrated the majority of responses from DMD students and PGR who disagreed with Question 2 about there being too many required vaccines, which was similar but higher among DMD students (65.2%) compared with PGRs (60.9%), χ^2^ = 0.279, d.f. = 1. *p* = 0.5972. In addition, some differences were found among those who disagreed with Question 4 about whether vaccines can make you sick (DMD, 28.7%; PGR 31.7%), χ^2^ = 0.161, d.f. = 1. *p* = 0.6881. Finally, fewer DMD students disagreed with Question 5 about whether vaccines are dangerous (43.7%) compared with PGRs (58.5%), χ^2^ = 3.199, d.f. = 1. *p* = 0.0737.

**Figure 1:**
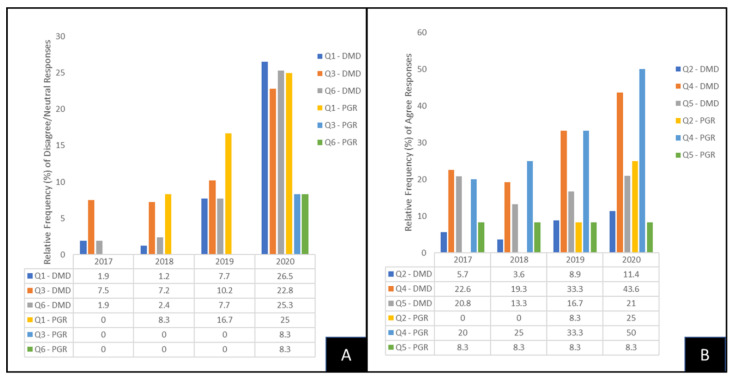
Graphic analysis of pro- and anti-vaccine response among DMD students and PG residents. (**A**) Relative frequency of disagree/neutral responses to pro-vaccines questions (Q1 Vaccines necessary; Q3 Vaccines safe; Q6 Vaccines effective) increased over time in both DMD and PGR cohorts. (**B**) Relative frequency of agree responses to anti-vaccine questions (Q2: Too many vaccines; Q4: Vaccines make you sick; also increased over time in both DMD and PGR cohorts, with increases observed among DMD but not PGR cohorts to Q5 (Vaccines dangerous).

To determine if any of the responses from DMD and PGRs to these important questions changed over time between the cohorts analyzed, these data were sorted and graphed by year (Figure 1). More specifically, the relative frequency of responses of DMD students and PGRs that did not agree with the pro-vaccine questions (Question 1—Vaccines necessary; Question 3—Vaccines safe; Question 6—Vaccines effective) were plotted and graphed (Figure 1A). These data demonstrated that the number of responses where DMD students disagreed/neutral to Question 1 (Vaccines necessary) increased over time (DMD 2017, 1.9% or n = 1/53; DMD 2018, 1.2% or n = 1/83; DMD 2019, 7.7% or n = 6/78; DMD 2020, 26.5% or n = 21/79), similar to PGR responses (PGR 2017, 0% or n = 0; PGR 2018, 8.3% or n = 1/12; PGR 2019, 16.7% or n = 2/23; PGR 2020, 25% or n = 3/12). In addition, DMD student survey responses with disagree/neutral to Question 3 (Vaccines safe) also increased over time (DMD 2017, 7.5% or n = 1/52; DMD 2018, 7.2% or n = 6/83; DMD 2019, 10.2% or n = 6/78; DMD 2020, 22.8% or n = 18/79) similar to PGR responses (PGR 2017, 0% or n = 0/5; PGR 2018, 0% or n = 0/12; PGR 2019, 0% or n = 0/12; PGR 2020, 8.3% or n = 1/12). Finally, DMD responses with disagree/neutral to Question 6 (Vaccine effective) also increased over time (DMD 2017, 1.9% or n = 1/53; DMD 2018, 2.4% or n = 2/83; DMD 2019, 7.7% or n = 6/78; DMD 2020, 25.3% n = 20/79), similar to PGR responses (PGR 2017, 0% or n = 0/5; PGR 2018, 0% or n = 0/12; PGR 2019, 0% or n = 0/12; PGR 2020, 8.3% or n = 1/12).

The relative frequencies of responses of DMD students and PGRs that agreed with the anti-vaccine questions (Question 2—Too many vaccines; Question 4—Vaccines make you sick; Question 5—Vaccines dangerous) were plotted and graphed (Figure 1B). These data demonstrated that the number of responses where DMD students agreed to Question 2 (Too many vaccines) increased over time (DMD 2017, 57% or n = 3/53; DMD 2018, 3.6% or n = 3/83; DMD 2019, 8.9% or n = 7/78; DMD 2020, 11.4% or n = 9/79), similar to PGR responses (PGR 2017, 0% or n = 0/5; PGR 2018, 0% or n = 0/12; PGR 2019, 8.3% or n = 1/12; PGR 2020, 25% or n = 3/12). In addition, DMD student survey responses with agree to Question 4 (Vaccines make you sick) also increased over time (DMD 2017, 22.6% or n = 12/53; DMD 2018, 19.3% or n = 16/83; DMD 2019, 33.3% or n = 26/78; DMD 2020, 43.6% n =34/79), similar to PGR responses (PGR 2017, 20% or n = 1/5; PGR 2018, 25% or n = 3/12; PGR 2019, 33.3% or n = 4/12; PGR 2020, 50% or n = 6/12). Finally, DMD responses with agree to Question 5 (Vaccines dangerous) also increased over time (DMD 2017, 20.8% or n = 11/53; DMD 2018, 13.3% or n = 11/83; DMD 2019, 16.7% or n = 12/78; DMD 2020, 26.5% or n = 21/79) but did not change among the PGR responses (PGR 2017, 8.3% or n = 1/12; PGR 2018, 8.3% or n = 1/12; PGR 2019, 8.3% or n = 1/12; PGR 2020, 8.3% or n = 1/12).

To assess HPV vaccine-related responses of DMD students and PGR, the survey responses to Questions 9–17 were compiled, and descriptive statistics calculated (Table 3). The analysis of these data demonstrated the majority of responses from DMD students and PGR who agreed with Question 9 about HPV vaccine awareness was similar but lower among DMD students (92.1%) compared with PGRs (95.1%), χ^2^ = 0.459, d.f. = 1, *p* = 0.4982. Similarly, small differences were observed among DMD students and PGR who agreed with Question 17 about receiving the HPV vaccine (DMD 37.9%, PGR 41.5%), χ^2^ = 0.195, d.f. = 1. *p* = 0.6588. In addition, some differences were also found among those who agreed with Question 14 about discussing the HPV vaccine with a doctor or physician (DMD, 37.5%; PGR 48.8%), χ^2^ = 1.911, d.f. = 1, *p* = 0.1669.

The analysis of these data demonstrated responses agreeing with Question 10 about the HPV vaccine being personally important, which was similar among DMD students (72.0%) and PGRs (70.0%), χ^2^ = 2.114, d.f. = 1. *p* = 0.1460. In addition, no significant differences were found among those who agreed with Question 15 about not having sufficient information about the HPV vaccine (DMD, 25.6%; PGR 26.8%), χ^2^ = 0.029, d.f. = 1. *p* = 0.8658. Finally, equal percentages of DMD students agreed with Question 16 about having concerns about HPV vaccine side effects (17.1%) compared with PGRs (17.1%), χ^2^ = 0.000, d.f. = 1. *p* = 0.9989.

**Figure 2:**
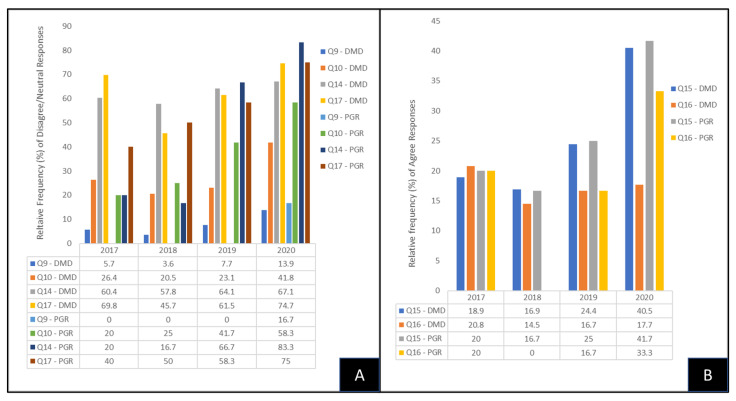
Graphic analysis of pro- and anti-HPV vaccine response among DMD students and PG residents. (**A**) Relative frequency of disagree/neutral responses to pro-HPV vaccines questions (Q9—HPV vaccine awareness; Q10—HPV vaccine important for me; Q14—Discussed HPV vaccines with doctor; Q17—Received HPV vaccine) increased over time in both DMD and PGR cohorts. (**B**) Relative frequency of agree responses to anti-HPV vaccine questions (Q15—Not enough information about HPV vaccine; Q16—Concerned about HPV vaccine side effects) also increased over time in both DMD and PGR cohorts.

To determine if any of the responses from DMD and PGRs to the HPV-related questions changed over time between the cohorts analyzed, these data were sorted and graphed by year (Figure 2). These data demonstrated that the relative frequency of responses where DMD students disagreed/neutral to Question 9 (aware of HPV vaccination) increased over time (DMD 2017, 5.7% or n = 3/53; DMD 2018, 3.6% or n = 3/83; DMD 2019, 7.7% or n = 6/78; DMD 2020, 13.9% or n = 11/79), similar to PGR responses (PGR 2017, 0% or n = 0/5; PGR 2018, 0% or n = 0/12; PGR 2019, 0% or n = 0/12; PGR 2020, 16.7% or n = 2/12) (Figure 2A). In addition, DMD student survey responses with disagree/neutral to Question 10 (HPV vaccination is important to me) also increased over time (DMD 2017, 26.4% or n = 14/53; DMD 2018, 20.5% or n = 17/83; DMD 2019, 23.1% or n = 18/78; DMD 2020, 41.8%, n = 33/79), similar to PGR responses (PGR 2017, 20% or n = 1/5; PGR 2018, 25% or n = 3/12; PGR 2019, 41.7% or 5/12; PGR 2020, 58.3% or n = 7/12). Finally, DMD responses with disagree/neutral to Question 14 (discussed HPV vaccine with doctor/physician) also increased over time (DMD 2017, 60.4% or n = 32/53; DMD 2018, 57.8% or n = 48/83; DMD 2019, 64.1% or n = 50/78; DMD 2020, 67.1% or n = 53/79), similar to PGR responses (PGR 2017, 20% or n = 1/5; PGR 2018, 16.7% or n = 2/12; PGR 2019, 66.7% or n = 8/12; PGR 2020, 83.3% or n = 10/12); this was also similar to those who disagreed with Question 17 (I have received the HPV vaccine) among the DMD students (DMD 2017, 69.8% or n = 37/53; DMD 2018, 45.7% or n = 38/83; DMD 2019, 61.5% or n = 48/78; DMD 2020, 74.7% or n = 59/79) and PGR response (PGR 2017, 40% or n = 2/5; PGR 2018, 50% or n = 6/12; PGR 2019, 58.3% or n = 7/12; PGR 2020, 75% or n = 9/12).

The relative frequency of responses of DMD students and PGRs that agreed with the anti-HPV vaccine questions was also sorted and graphed to determine changes over time (Figure 2B). These data demonstrated that the number of responses where DMD students agreed to Question 15 (Not enough information about HPV vaccine) increased over time (DMD 2017, 18.9% or n = 10/53; DMD 2018, 16.9% or 14/83; DMD 2019, 24.4% or n = 19/78; DMD 2020, 40.5% or n = 32/79), similar to PGR responses (PGR 2017, 20% or n = 1/5; PGR 2018, 16.7% or n = 2/12; PGR 2019, 25% or n = 3/12; PGR 2020, 41.7% or n = 5/12). In addition, DMD student survey responses with agree to Question 16 (Concerned about HPV vaccine side effects) also increased over time (DMD 2017, 20.8% or n = 11/53; DMD 2018, 14.5% or n = 12/83; DMD 2019, 16.7% or n = 13/78; DMD 2020, 17.7% or n = 14/79), which was similar to PGR responses (PGR 2017, 20% or n = 1/5; PGR 2018, 0% or n = 0/12; PGR 2019, 16.7% or 2/12; PGR 2020, 33.3% or n = 4/12).

## 4. Discussion

The primary goal of this study was to provide a more current and up-to-date evaluation of recent trends in vaccine awareness, knowledge, and beliefs among dental students and postgraduate residents, which might suggest opportunities for educational interventions or training. The results of this study clearly identify several recent changes that may signal important trends in attitudes and beliefs regarding vaccination. For example, although most of the respondents reported they believe vaccines are necessary, safe, and effective, a growing percentage of both DMD students and PG residents over the past 4 years indicated negative attitudes and responses to all three questions. This represents a significant change in attitudes and beliefs regarding vaccination since the first collection and analysis of data from these populations [26]. Moreover, these data may represent part of a larger and more pervasive trend in vaccine hesitancy and attitude shifts among healthcare workers and students based upon social media and cultural misinformation that have been recently reported in other studies [31,32].

These findings also suggest more of these respondents now believe there are too many required vaccines, with significant percentages reporting they believe vaccination can make you sick or may be dangerous. These data support observations in other recent studies that suggest other healthcare workers and students are also experiencing significant negative shifts towards vaccine hesitancy, in particular due to self-reported concerns about safety and efficacy that are not based upon peer-reviewed evidence [33,34]. In fact, a growing body of evidence now suggests that vaccine hesitancy may be increasing globally, even among highly educated healthcare workers, which is a cause for concern due the large number of vaccine preventable illnesses that require high levels of vaccine acceptance and compliance for herd immunity [35,36].

An additional finding of note is the growing percentage of respondents in this study with divergent responses found with respect to HPV vaccination. More specifically, although most students and residents reported being aware of HPV vaccination, a large shift in responses indicated that more respondents felt they had insufficient knowledge and information about the HPV vaccine specifically, which corresponded with a decrease in personal importance of HPV vaccination. This information corresponds with and supports the findings of other recent research that describes an overall increase in the number of people (and providers) who feel they have inadequate knowledge and insufficient information about the HPV vaccine to make clear, rational decisions for their children based upon evidence [37,38,39].

Although this study highlights important findings that may suggest more targeted interventions and curricular training are needed to provide answers to questions about vaccine hesitancy and improve scientific literacy and knowledge about vaccine safety and efficacy, there are limitations of this study that should be considered. One of the most important of these is the study population, which consisted of highly educated graduate-level students and postgraduate residents. However, although vaccine hesitancy may be higher among the general population, the growing trend of vaccine hesitancy among healthcare workers and graduate healthcare students suggests that more active engagement and direct interventions may be necessary to connect all segments of the population with scientific knowledge and evidence-based information [40,41]. Another important consideration is the retrospective nature of this study, which has analyzed previously collected data and was not able to provide more in-depth information about socioeconomic factors or socio-cultural information that might provide more opportunities for evaluating and assessing the causes for these shifts in vaccination beliefs and attitudes [42,43].

## 5. Conclusions

Despite the limitations inherent in this type of research, these data provide novel insights into the recent changes in attitudes, awareness, and beliefs regarding vaccination among graduate-level dental students and postgraduate residents. Moreover, careful analysis of these shifts in attitudes and knowledge about HPV vaccination suggests that curricular integration of vaccine research and other research about vaccine hesitancy may be needed to answer questions about vaccine safety, side effects, and efficacy in a supportive learning environment that fosters critical thinking and evidence-based practice and decision making.

## Figures and Tables

**Table 1:** Demographic analysis of DMD/PGR cohorts and survey respondents.

Demographic	Study Sample	Cohorts	Statistical Analysis
Sex Male Female	DMD study sample 54.9% (n = 161/293) 45.1% (n = 132/293)	DMD cohorts 57.8% (n = 189/327) 42.2% (n = 138/327)	χ^2^ = 0.510, d.f. = 1 *p* = 0.4750
Race/Ethnicity White Asian/Pacific Islander Hispanic Black/Other	DMD study sample 41.6% (n = 122/293) 47.4% (n = 139/293) 9.6% (n = 28/293) 1.4% (n = 4/293)	DMD cohorts 42.8% (n = 140/327) 46.8% (n = 153/327) 8.6% (n = 28/327) 1.8% (n = 6/327)	χ^2^ = 0.087, d.f. = 1 *p* = 0.7674
Age Average age	25.46 years	25.62 years	Students *t*-test *p* = 0.7134
Total number of responses per cohort	2017: 67.9% (n = 53/78) 2018: 97.6% (n = 83/85) 2019: 95.1% (n = 78/82) 2020: 96.3% (n = 79/82)		
Sex Male Female	PG study sample 46.3% (n = 19/41) 53.7% (n = 22/41)	PG cohorts 45.8% (n = 22/48) 54.2% (n = 26/48)	χ^2^ = 0.002, d.f. = 1 *p* = 0.9618
Race/Ethnicity White Asian/Pacific Islander Hispanic Black/Other	PG study sample 51.2% (n = 21/41) 46.3% (n = 19/41) 0.0% (n = 0/41) 2.4% (n = 1/41)	PG cohorts 50.0% (n = 24/48) 47.9% (n = 23/48) 0.0% (n = 0/48) 2.1% (n = 1/48)	χ^2^ = 0.013, d.f. = 1 *p* = 0.9087
Age Average age	27.1 years	26.7 years	Students *t*-test *p* = 0.7749
Total number of responses per cohort	2017: 41.6% (n = 5/12) 2018: 100% (n = 12/12) 2019: 100% (n = 12/12) 2020: 100% (n = 12/12)		

Key: dental student (DMD), postgraduate resident (PGR), n.r. = no responses.

**Table 2:** General vaccine related survey responses of DMD students and PGR.

Question	Agree	Neutral	Disagree	Not Applicable
1. Vaccines are necessary to protect public health	DMD 90.1% PGR 85.4% (DMD = 264/293) (PGR = 35/41)	DMD 2.7% PGR 0% (DMD = 8/293) (PGR = 0/41)	DMD 7.2% PGR 14.6% (n = 21/293) (n = 6/41)	n.r.
2. There are too many required vaccines	DMD 7.5% PGR 9.8% (DMD = 22/293) (PGR = 4/41)	DMD 27.3% PGR 29.3% (DMD = 80/293) (PGR = 12/41)	DMD 65.2% PGR 60.9% (n = 191/293) (n = 25/41)	n.r.
3. Vaccines are generally safe	DMD 87.7% PGR 97.6% (DMD = 257/293) (PGR = 40/41)	DMD 11.9% PGR 2.4% (DMD = 35/293) (PGR = 1/41)	DMD 0.3% PGR 0% (n = 1/293) (n = 0/41)	n.r.
4. Vaccination can make you sick	DMD 30.0% PGR 34.1% (DMD = 88/293) (PGR = 14/41)	DMD 41.3% PGR 34.1% (DMD = 121/293) (PGR = 14/41)	DMD 28.7% PGR 31.7% (n = 84/293) (n = 13/41)	n.r.
5. Some vaccines are dangerous	DMD 19.1% PGR 9.8% (DMD = 56/293) (PGR = 4/41)	DMD 37.2% PGR 31.7% (DMD = 109/293) (PGR = 13/41)	DMD 43.7% PGR 58.5% (n = 128/293) (n = 24/41)	n.r.
6. Vaccines are generally effective	DMD 90.1% PGR 97.6% (DMD = 264/293) (PGR = 40/41)	DMD 2.4% PGR 2.4% (DMD = 7/293) (PGR = 1/41)	DMD 7.5% PGR 0% (n = 22/293) (n = 0/41)	n.r.
7. I follow the ACIP vaccine guidelines for myself	DMD 80.9% PGR 80.5% (DMD = 237/293) (PGR = 33/41)	DMD 19.9% PGR 0% (DMD = 29/293) (PGR = 0/41)	DMD 9.2% PGR 19.5% (n = 27/293) (n = 8/41)	n.r.
8. I adhere to the vaccine guidelines for my family	DMD 63.5% PGR 73.2% (DMD = 186/293) (PGR = 30/41)	DMD 8.2% PGR 0% (DMD = 24/293) (PGR = 0/41)	DMD 2.7% PGR 0% (n = 8/293) (n = 0/41)	DMD 25.6% PGR 26.8% (n = 75/293) (n = 11/41)

Key: dental student (DMD), postgraduate resident (PGR), n.r. = no responses.

**Table 3:** HPV vaccine related survey responses of DMD students and PGR.

Question	Agree	Neutral	Disagree	Not Applicable
9. I am aware of a vaccine for human papillomavirus (HPV)	DMD 92.1% PGR 95.1% (DMD = 270/293) (PGR = 39/41)	DMD 5.5% PGR 2.4% (DMD = 16/293) (PGR = 1/41)	DMD 2.4% PGR 2.4% (n = 7/293) (n = 1/41)	n.r.
10. HPV vaccination is important for me	DMD 72.0% PGR 70.0% (DMD = 211/293) (PGR = 25/41)	DMD 23.2% PGR 34.1% (DMD = 68/293) (PGR = 14/41)	DMD 4.8% PGR 4.9% (n = 14/293) (n = 2/41)	n.r.
11. HPV vaccination is important for (my) spouse/partner	DMD 58.0% PGR 65.6% (DMD = 170/293) (PGR = 27/41)	DMD 17.4% PGR 0% (DMD = 51/293) (PGR = 0/41)	DMD 3.1% PGR 24.4% (n = 9/293) (n = 10/41)	DMD 21.5% PGR 9.8% (n = 63/293) (n = 4/41)
12. HPV vaccination is important for (my) daughter(s)	DMD 47.8% PGR 58.5% (DMD = 140/293) (PGR = 24/41)	DMD 7.2% PGR 0% (DMD = 21/293) (PGR = 0/41)	DMD 1.0% PGR 12.2% (n = 3/293) (n = 5/41)	DMD 44% PGR 29.3% (n = 129/293) (n = 12/41)
13. HPV vaccination is important for (my) son(s)	DMD 43.7% PGR 48.8% (DMD = 128/293) (PGR = 20/41)	DMD 12.6% PGR 0% (DMD = 37/293) (PGR = 0/41)	DMD 3.8% PGR 21.0% (n = 11/293) (n = 9/41)	DMD 40% PGR 29.3% (n = 117/293) (n = 12/41)
14. I have discussed HPV vaccination with a doctor	DMD 37.5% PGR 48.8% (DMD = 110/293) (PGR = 20/41)	DMD 6.8% PGR 12.2% (DMD = 20/293) (PGR = 5/41)	DMD 55.6% PGR 39.0% (n = 163/293) (n = 16/41)	n.r.
15. I do not have enough information about the HPV vaccine	DMD 25.6% PGR 26.8% (DMD = 75/293) (PGR = 11/41)	DMD 29.0% PGR 29.3% (DMD = 85/293) (PGR = 12/41)	DMD 45.4% PGR 43.9% (n = 133/293) (n = 18/41)	n.r.
16. I am concerned about possible HPV vaccine side effects	DMD 17.1% PGR 17.1% (DMD = 50/293) (PGR = 7/41)	DMD 31.1% PGR 34.1% (DMD = 91/293) (PGR = 14/41)	DMD 51.9% PGR 48.8% (n = 152/293) (n = 20/41)	n.r.
17. I have already received the HPV vaccine	DMD 37.9% PGR 41.5% (DMD = 111/293) (PGR = 17/41)	n.r.	DMD 62.1% PGR 58.5% (n = 182/293) (n = 24/41)	n.r.

Key: dental student (DMD), postgraduate resident (PGR), n.r. = no responses.

## Data Availability

The data presented in this study are available on request from the corresponding author. The data are not publicly available due to the study protocol data protection parameters requested by the IRB and OPRS for the initial study approval.
